# Reduced visitation to buzz‐pollinated *Cyanella hyacinthoides* in the presence of other pollen sources in the hyperdiverse Cape Floristic Region

**DOI:** 10.1002/ece3.8784

**Published:** 2022-04-02

**Authors:** Jurene E. Kemp, Francismeire J. Telles, Mario Vallejo‐Marín

**Affiliations:** ^1^ 7622 Biological and Environmental Sciences University of Stirling Stirling UK; ^2^ 28119 Programa de Pós‐Graduação em Ecologia e Conservação de Recursos Naturais Universidade Federal de Uberlândia Uberlândia MG Brazil

**Keywords:** bee vision, community ecology, fynbos, interspecific competition, reproductive ecology, sonication

## Abstract

Many plant species have floral morphologies that restrict access to floral resources, such as pollen or nectar, and only a subset of floral visitors can perform the handling behaviors required to extract restricted resources. Due to the time and energy required to extract resources from morphologically complex flowers, these plant species potentially compete for pollinators with co‐flowering plants that have more easily accessible resources. A widespread floral mechanism restricting access to pollen is the presence of tubular anthers that open through small pores or slits (poricidal anthers). Some bees have evolved the capacity to remove pollen from poricidal anthers using vibrations, giving rise to the phenomenon of buzz‐pollination. These bee vibrations that are produced for pollen extraction are presumably energetically costly, and to date, few studies have investigated whether buzz‐pollinated flowers may be at a disadvantage when competing for pollinators’ attention with plant species that present unrestricted pollen resources. Here, we studied *Cyanella hyacinthoides* (Tecophilaeaceae), a geophyte with poricidal anthers in the hyperdiverse Cape Floristic Region of South Africa, to assess how the composition and relative abundance of flowers with easily accessible pollen affect bee visitation to a buzz‐pollinated plant. We found that the number of pollinator species of *C. hyacinthoides* was not influenced by community composition. However, visitation rates to *C*. *hyacinthoides* were reduced when the relative abundances of flowers with more accessible resources were high. Visitation rates were strongly associated with petal color, showing that flower color is important in mediating these interactions. We conclude that buzz‐pollinated plants might be at a competitive disadvantage when many easily accessible pollen sources are available, particularly when competitor species share its floral signals.

## Graphical abstract

Due to the time and energy required to extract resources from morphologically complex flowers, these plant species potentially compete for pollinators with co‐flowering plants that have more easily accessible resources. We show that buzz‐pollinated plants might be at a competitive disadvantage when many easily accessible pollen sources are available, particularly when competitor species share its floral signals.

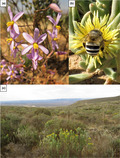

## 1 INTRODUCTION

The majority of flowering plants are pollinated by animals (Ollerton et al., 2011; Rodger et al., 2021), and most animal‐pollinated species offer resources such as pollen, nectar, oils, and scents as rewards to attract floral visitors. The degree to which these resources are accessible to floral visitors varies between plant species, and the accessibility of resources is often modulated through morphological restrictions, such as nectar tubes or keel flowers (Córdoba & Cocucci, 2011; Santamaría & Rodríguez‐Gironés, 2015). Although morphological restrictions can limit or prevent inefficient pollen vectors or resource thieves from gaining access to floral resources (Santamaría & Rodríguez‐Gironés, 2015; van der Kooi et al., 2021), these barriers can also influence visitation by efficient pollinators, particularly when floral resources can more easily be obtained from flowers that do not restrict access to resources.

Flowers with morphologies that require complex handling behaviors (i.e., requiring integration of multiple individual tasks, and often resulting in high energy or time requirements) for resource extraction co‐occur and potentially compete with flowers that offer more easily accessible resources. Floral visitors that have the ability to extract resources from morphologically complex flowers may preferentially visit complex flowers either when these flowers offer larger resource quantities or higher quality resources than flowers with unrestricted resources (Arroyo & Dafni, 1995; Warren & Diaz, 2001), or when the probability of obtaining resources is higher in complex flowers because few other species can access the resources (Warren & Diaz, 2001). Alternately, if flowers with more easily accessible resources are abundant in a community, the costs associated with learning to handle complex flowers, as well as the time and energy costs of foraging on complex flowers, might result in lower visitation to complex flowers if similar rewards are offered by flowers with unrestricted resources (Lázaro et al., 2013; McCall & Primack, 1992; Zhao et al., 2016). These contrasting effects of restricting access to floral resources have been observed in multiple studies, where some work has reported floral visitors favoring complex flowers (Stout et al., 1998) and others have found that floral visitors prefer flowers where resources can be accessed more easily (Kunin & Iwasa, 1996; Lázaro et al., 2013; McCall & Primack, 1992; Stout et al., 1998). The choices of floral visitors in communities where resources are available in a range of restrictions levels are likely contingent on (A) the identity of competitor species and the differences in resource quality and quantity between species (Stout et al., 1998), (B) the abundances of different plant species (Kunin & Iwasa, 1996), and (C) the degree of floral trait overlap between plant species which likely influences attraction to plant species (Hargreaves et al., 2009; Lázaro et al., 2013). Thus, plant species that restrict access to resources can potentially be at a competitive disadvantage under certain conditions (as mentioned above), and this is likely contingent on the abundance of unrestricted resources offered by the co‐flowering community, as well as the degree of floral trait overlap.

One way in which plants that offer pollen as primary reward can restrict access to pollen grains is through poricidal anthers (Buchmann, 1983; van der Kooi et al., 2021). Some species of bees have evolved the capacity to produce vibrations (also known as floral vibrations or sonication) that facilitate the removal of pollen grains from poricidal anthers (Buchmann, 1983; De Luca & Vallejo‐Marín, 2013). The interaction between plants with specialized floral morphologies, such as poricidal anthers, and the bee behavior of deploying floral vibrations has given rise to the phenomenon of buzz‐pollination (Buchmann, 1983; Vallejo‐Marín, 2019). During buzz‐pollination, bees typically grasp the anthers with their mandibles, curl their bodies around the anthers, and then generate vibrations that result in pollen being released from the anthers through apical slits or pores (De Luca & Vallejo‐Marín, 2013). Using vibrations for pollen extraction is likely energetically expensive, as the production of floral vibrations by bees involves rapid contraction of the same thoracic muscles that power energetically costly wingbeat during flight (King & Buchmann, 2003). During flight, these muscles consume as much as 100 times the energy than the resting metabolic rate (Dudley, 2002). Floral vibrations have higher frequency and amplitude (velocity, acceleration, and displacement) than flight vibrations (Pritchard & Vallejo‐Marín, 2020) and, therefore, it is likely that floral vibrations are equally or more energetically costly as those produced during flight. Because of the energetic costs associated with vibratile pollen extraction, we might expect bees to favor more easily accessible pollen resources under certain circumstances. Buzz‐pollination is prevalent among both plants (6%–8% of all plant species across 65 families (Buchmann, 1983) and bees (15% of bee genera containing 58% of bee species (Cardinal et al., 2018)), however, our understanding of how visitation to buzz‐pollinated plants is influenced by the availability of unrestricted pollen resources in the surrounding plant community is limited.

Recent work has shown that competition for pollination services between buzz‐pollinated individuals is prevalent (Mesquita‐Neto et al., 2018; Soares et al., 2021). It is likely that these buzz‐pollination interactions are also influenced by the presence of non‐poricidal taxa with unrestricted pollen resources. We hypothesize that if the energetic and, potentially, learning costs (per unit resource acquired) for bees (Laverty, 1980; Russell et al., 2016) are higher for flowers requiring vibratile pollen extraction than unrestricted flowers, then visitation to plants with poricidal anthers should be reduced when flowers that do not restrict access to pollen are available in high relative abundances in a community. An alternative hypothesis is that because only a subset of floral visitors in a community can use vibrations to extract pollen, plants with poricidal anthers could potentially act as a private and reliable pollen resource to particular bee species. If the benefits of reduced search time and energy associated with collecting pollen from a reliable resource outweigh the pollen extraction costs, then we might expect consistent visitation from these bees to buzz‐pollinated flowers regardless of community context.

The Cape Floristic Region (CFR) of South Africa is well‐suited for studying the effects of variation in co‐flowering species composition on a focal plant species due to the sharp spatial and temporal changes in the composition of flowering communities (Cowling, 1992; Simmons & Cowling, 1996). Our study focuses on buzz‐pollinated *Cyanella hyacinthoides* (Tecophilaeaceae) in the CFR. This species is widespread and thus co‐occurs with a variety of other plant species, making it ideal to study the effects of co‐flowering species composition on pollinator visitation. *Cyanella hyacinthoides* is a cormous geophyte endemic to the CFR that flowers during Austral spring (August to November) (Manning & Goldblatt, 2012). It has light blue flowers with six poricidal anthers that vary in morphology (five smaller upper anthers and one larger lower anther) (Dulberger & Ornduff, 1980). Plants from this species can present multiple inflorescences and each inflorescence can produce up to 15 flowers. Individual flowers can remain open for six or seven days if not pollinated (Dulberger & Ornduff, 1980), but flowers close within a few hours after pollination has occurred (personal observation). Self‐compatibility varies between populations, with two‐thirds of assessed populations exhibiting complete self‐incompatibility (Dulberger & Ornduff, 1980), indicating that the persistence of this species mostly relies on successful pollinator‐mediated reproduction.

Here, we contrast how visitation rates by bees that can successfully manipulate the buzz‐pollinated *C*. *hyacinthoides* is influenced by the availability of more easily accessible resources, that is, co‐occurring plant species with simple floral morphologies. We predict that if *C*. *hyacinthoides* competes with flowers with unrestricted pollen for pollination services, there should be a reduction in the number of pollinator species and their visitation rates to *C*. *hyacinthoides* when the relative abundances of flowers with unrestricted pollen resources are high. Alternatively, if *C*. *hyacinthoides* is a private and reliable pollen resource to bee species that can successfully manipulate poricidal anthers (thus reducing the time and energy searching for flowers that contain pollen), we expect consistent visitation from these bees regardless of community context. Further, because floral traits can influence pollinator choices, we evaluated whether the pollinators of *C*. *hyacinthoides* were preferentially visiting flowers with particular floral traits. Our study addresses the following questions: (1) How does the number of species visiting *C*. *hyacinthoides* vary when the relative availability of unrestricted rewards changes? (2) How do visitation rates to *C*. *hyacinthoides* vary when the relative availability of unrestricted pollen resources changes? (3) Which floral traits modulate visitation by the pollinators *of C*. *hyacinthoides*?

## 2 METHODS

### 2.1 Sampling sites

**FIGURE 1:**
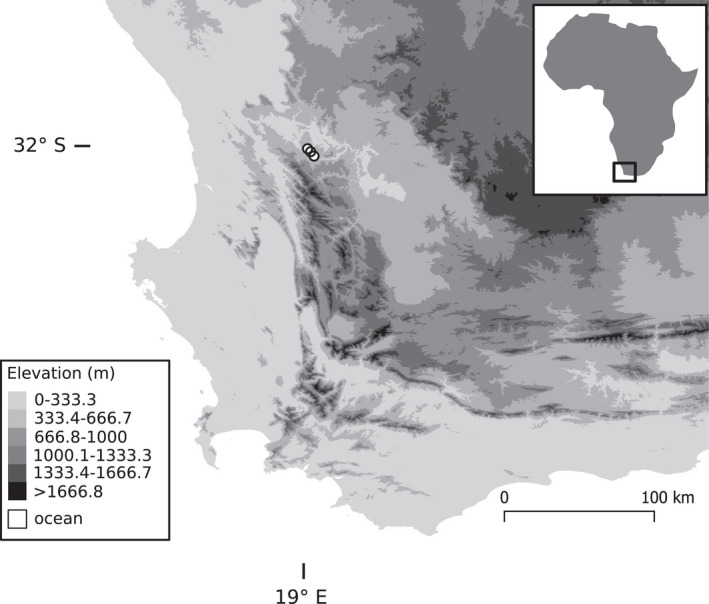
Elevation map of south‐western South Africa, which primarily contains the hyperdiverse Cape Floristic Region. The sampling sites were located in the northern Cederberg mountain range, indicated by white circles

We conducted our study in the Pakhuis Pass region of the northern Cederberg mountain range, situated in the west of the CFR in September‐October 2019 (Figure 1). This winter‐rainfall area receives an annual rainfall of 270 ± 60 mm (mean ± SD; Pauw and Stanway (2015)). Most of the flowering in this region occurs during late‐winter/early‐spring (late‐July to early‐September), and flowering quickly ends when temperatures increase (Pauw & Stanway, 2015). Buzz‐pollinated species in this region generally start flowering in September, and flowering continues throughout summer after most other flowering has ended (Manning & Goldblatt, 2012). The Cederberg region is home to multiple buzz‐pollinated species, including *Cyanella hyacinthoides* (Tecophilaeaceae), *C*. *orchidiformis*, *C*. *alba*, *Ixia scillaris* (Iridaceae), *Solanum tomentosum* (Solanaceae), *Chironia linoides* (Gentianaceae), *C*. *baccifera*, and *Roridula dentata* (Roridulaceae), amongst others. For this study, we focused on communities containing *Cyanella hyacinthoides* as it is widespread and occurs among a wide variety of co‐flowering species.

**FIGURE 2:**
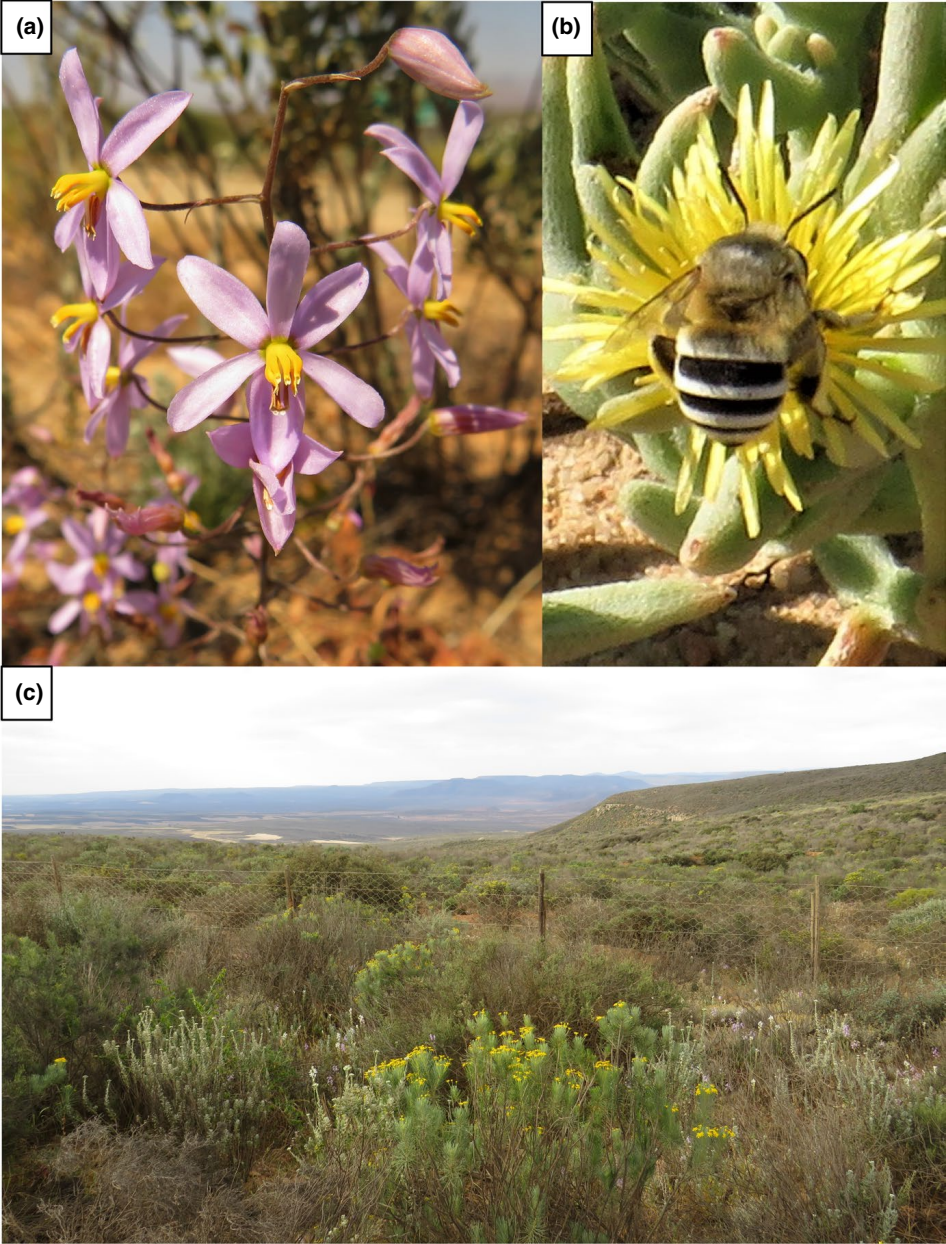
*Cyanella hyacinthoides*, its main pollinator, and the vegetation type of the sites are shown. (a) *C*. *hyacinthoides* is a cormous perennial that presents flowers with poricidal anthers on multiple inflorescences. Flowers are approximately 16 mm in diameter across the longest axis. (b) *Amegilla* cf. *niveata* was the most frequent visitor to *C*. *hyacinthoides* in these communities. In this photo, a female is drinking nectar from a *Cleretum* flower (flower approximately 16 mm in diameter). (c) The sites were situated in Rainshadow Valley Karoo shrubland. The shrub in the foreground with yellow flowers is approximately 80 cm tall. Photos: JEK

We identified three sites where *C*. *hyacinthoides* was abundant. The sites present a shrubland vegetation that is classified as Rainshadow Valley Karoo (Mucina et al., 2006), located in the transition between fynbos and the drier inland vegetation (Figure 2). Each site covered approximately 100 m × 100 m, and the sites were separated by 4–10 km. We exploited the CFR’s tendency to sharp temporal turnover in community composition, and we specifically chose sites where (A) few individuals of *C*. *hyacinthoides* recently started flowering and many individuals had not yet started flowering (i.e., *C*. *hyacinthoides* would continue to flower for at least two to three more weeks), (B) multiple plant species with unrestricted pollen resources were at a late stage of flowering (i.e., few buds and many seed pods present, and flowering would end within a week), and (C) where multiple plant species with unrestricted pollen resources were still in bud stage (i.e., would start flowering in about a week). These communities thus showed a change in *C*. *hyacinthoides* flower abundances over a short timeframe as more individuals start flowering, as well as a change in the composition of other plant species as the late‐stage flowering species (B) end their flowering and the bud‐stage flowers (C) initiate flowering (see *Results* for details on plant species turnover). By sampling each of these three sites twice, we were able to observe the pollination interactions of *C*. *hyacinthoides* in different community contexts, whilst controlling for environmental and site‐specific factors. To verify that the communities showed sufficient spatial and temporal variation in flower composition, we quantified plant turnover by calculating beta diversity (Horn similarity, following Jost (2007)).

### 2.2 Pollinator observations

For each of the three sites, two observers (J.E.K. and F.J.T.) recorded interactions over 3–5 days, depending on the weather. Each site was sampled a second time, approximately 9 days after the first sampling session ended. This resulted in the six data sets that we consider as six communities (see *Results* for turnover in plant composition between sampling sessions). The first site was sampled from 17 to 22 September 2019, and resampled from 1 to 4 October 2019. The second site was sampled 22–25 September 2019, and resampled 4–6 October 2019. The third site was sampled 25–28 September 2019, and resampled 8–12 October 2019.

Floral visitation observations commenced when bee activity started, that is, after 06:00 h, depending on the weather. If the temperature exceeded 30°C and bee activity decreased, observations were halted and resumed later in the day. Interactions between bees and flowers were recorded in 20‐min intervals, and multiple plant species were observed simultaneously. During each 20‐min interval, a 1–4 m^2^ patch of flowers was observed (patch size depended on flower densities) by each of the two observers (following Pauw and Stanway (2015) whom previously constructed interaction networks in this region). Patches of flowers were observed from a distance of 0.5–2 m. We recorded the number of flowers visited by each bee species in a patch, and for compound inflorescences (such as those of Compositae), we recorded the number of visits per inflorescence. Patches for recording flower visits were chosen to optimize the number of flowers recorded per observation period. We also chose patches that included species that occurred in low abundances at the site. To account for our unequal sampling effort, we calculated the visitation rate to each plant species by each bee species as visits per flower per 20 min (Aizen et al., 2008; Pauw & Stanway, 2015; Vázquez et al., 2005). We multiplied the visits/flower/20 min with 1000 and rounded the result to create integers, thus calculating visits per 1000 flowers per 20 min. We did this as some of our analyses required integers as input. In total, interactions were observed for 607 20‐min intervals, resulting in 202.3 observation hours.

To quantify plant species densities at each site for each sampling session, we counted the number of flowers in 25–30 randomly placed 4 m^2^ plots at each site. The recording of plant species densities was temporally spread out across the 3–4 days that pollinator observations were done at a site. Our analyses (described below) relied on the interaction frequencies of vibrating bees to all plant species in the community from which they were observed to collect pollen. We (M.V.M. and J.E.K.) thus identified the vibrating bees to genus or species‐level using the keys by Eardley (1994) and Eardley and Brooks (1989). The plant species that were visited by the vibrating bees were identified to species level using Manning and Goldblatt (2012). We did not identify the additional bee and plant species to species‐level, however, we identified the additional bee species as morphospecies in the field (through capture, behavior, and photographs). The additional plant species were identified to genus‐level, and assigned to a morphospecies. Although our identification of some plant and bee species occurred at a higher taxonomic level, all data recording occurred at the (morpho)species level. Plant and insect species samples are housed at the University of Stirling (UK).

#### 2.2.1 How does the number of species visiting *C. hyacinthoides* change when the availability of unrestricted resources changes?

The number of bee species visiting a plant species (i.e., ecological pollination specialization—*sensu* Armbruster (2017)) can be described by multiple metrics, and we calculated two metrics that captured this within each community. The first metric is *interaction partner richness*, which measures the raw number of bee species that visits and vibrates *C*. *hyacinthoides*. For instance, if five species visit and vibrate *C*. *hyacinthoides*, then the interaction partner richness would be 5. The second metric is *interaction partner diversity*, calculated using Hill numbers of the Shannon diversity index (Jost, 2007). This metric calculates the number of bee species that visits and vibrate *C*. *hyacinthoides* and weights it with the interaction frequency of each pollinator species, thus accounting for interaction evenness (Kemp et al., 2019). Thus, if *C*. *hyacinthoides* is visited by many insect species, but only a few of these species have high visitation rates to *C*. *hyacinthoides*, this metric will indicate that *C*. *hyacinthoides* is effectively visited by few pollinator species. For instance, if one pollinator species makes 10 visits to *C*. *hyacinthoides* and four species each make one visit, the interaction partner diversity would be 2.70. This metric thus adjusts for uneven visitation by pollinators.

**FIGURE 3:**
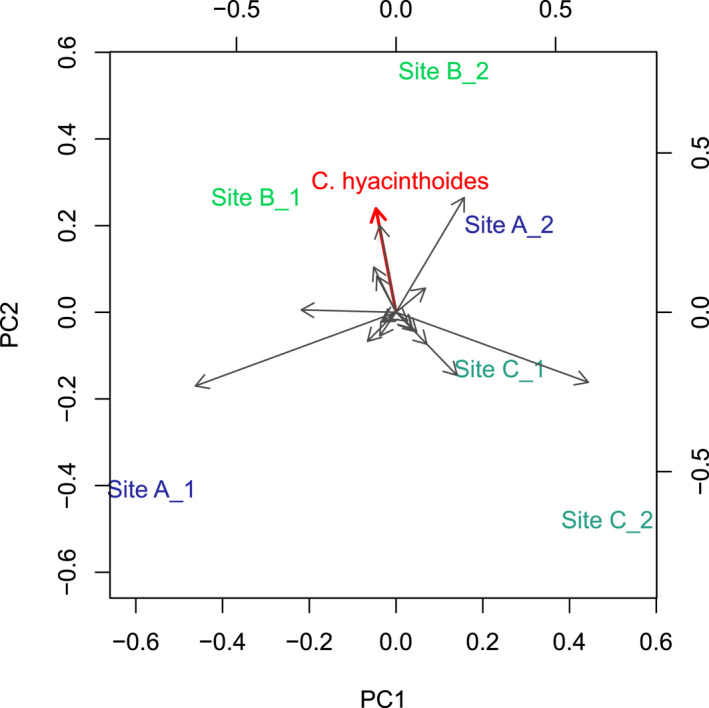
Principal components analysis of pollen‐offering plant species community composition based on plant densities per m^2^. Three sites were targeted for sampling, and each site was sampled twice. The plant community composition of the six communities are indicated on the plot, with communities sampled from the same site indicated by the same color. The first PC explained 49% of the variation in plant community composition, and the second PC explained 26% of the variation in plant community composition. Variation in *C*. *hyacinthoides* abundances were associated with PC2. Arrows indicate the association between different plant species and the principal components, with *C*. *hyacinthoides* indicated in red

To assess whether ecological specialization of *C*. *hyacinthoides*, as measured by the two metrics created above changes when the surrounding community composition changes, we first reduced community composition into a single variable by conducting a principal component analysis (PCA). Prior to the PCA, plant abundance data were Hellinger transformed using the “vegan” package (Oksanen et al., 2019) in R (R core team, 2020), as recommend for community data with many zeros (Legendre & Gallagher, 2001). We conducted a PCA of the plant densities per m^2^ for each community using the “prcomp” function in R (R core team, 2020). All pollen‐offering plant species were included in the PCA (Figure 3). The first principal component (PC) explained 49% of variation, and the second PC explained 26% of variation. High positive values along the second axis were associated with high *C*. *hyacinthoides* (loading PC2: 0.45) abundances. We thus used this second principal component as proxy for community composition in our subsequent analyses as we were interested in variation associated with *C*. *hyacinthoides*. Next, we calculated the total number of visits that vibrating bees made to all pollen‐offering plants in a community. This was calculated by multiplying the visits per flower with the flower density per m^2^, and then multiplying the result by 10,000 m^2^ (approximate community size), giving the number of visits each vibrating bee species made to each plant species. Visits were summed across plant species to give the total number of visits made by these bee species within a community, and we used this as proxy for the abundances of vibrating bees.

We analyzed two statistical models with the specialization metrics as response variables. Vibrating bee abundances and community composition (as represented by PC2 from the PCA above) as calculated above were used as explanatory variables. Specifically, Poisson regression was used to test the influence of vibrating bee abundances (natural log‐transformed to improve model fit) and community composition on interaction partner richness. Further, we conducted a linear regression to assess the influence of vibrating bee abundances (natural log‐transformed to improve model fit) and community composition on interaction partner diversity.

#### 2.2.2 How do visitation rates to *C. hyacinthoides* change when the relative availability of unrestricted resources changes?

We implemented a multistep approach to test whether visitation rates of vibrating bees to *C*. *hyacinthoides* is associated with the relative availability of unrestricted pollen resources in the surrounding community. First, we accounted for the variation in bee abundances between the six communities as this could directly influence visitation rates. To do this, we conducted a Poisson regression with visitation rate to *C*. *hyacinthoides* as response variable and vibrating bee abundances as predictor variable (estimate = 0.0659, *z*‐value = 2.319, *p* = .02). The residuals from this analysis were extracted and used in subsequent analyses, and these residuals represented visitation rates of vibrating bees to *C*. *hyacinthoides* after accounting for vibrating bee abundances. Second, we accounted for site effects seeing that each site was sampled twice. To do this, we first attempted a mixed effect model with the visitation rate residuals as response variable, the community composition PC2 as predictor variable, and site as random factor. However, due to our low sample size, the model was singular and could thus not be used. As an alternative approach, we conducted a paired t‐test with visitation rate residuals as response variable and site as grouping factor. For the predictor variable, each community was classified as either “High *C*. *hyacinthoides* relative abundances” or “Low *C*. *hyacinthoides* relative abundances” based on their position along PC2. The classification was made by assigning one of the two communities sampled at each site to one of the two categories. For instance, community A1 was classified as “Low *C*. *hyacinthoides* relative abundances” and community A2 as “High *C*. *hyacinthoides* relative abundances” due to their relative positions along PC2 (see Figure 3).

The paired *t*‐test thus tested whether visitation rates to *C*. *hyacinthoides* (after accounting for bee abundances) differed within a site when the relative abundances of *C*. *hyacinthoides* differed.

#### 2.2.3 Which floral traits modulate visitation by the pollinators of *C. hyacinthoides*?

For each plant species, we measured the plant height and flower diameter (along its longest dimension for asymmetric flowers). For this, we measured a median of 30 flowers per species. Measurements were spread out across the three to four days in which observations were done at a site. Additionally, we recorded flower color by measuring reflectance spectra indoors at a 45° angle to the petal surface with an OceanOptics USB4000 Spectrometer (Ocean Optics, Dunedin, FL, USA) calibrated with a diffuse reflectance WS‐2 white standard. Multiple measurements were taken per species (mean = 14, range = 6–30), and these were averaged to obtain a single spectrogram per species. We modelled these spectra in honeybee vision using Chittka's hexagon model (Chittka, 1992), assuming a D65 illumination and a standard green background, in the “pavo” package (Maia et al., 2019) in R. We chose the honeybee visual model because the specifics of the photoreceptors of most of the bee species in this study are unknown, except for honeybees. Plant species were subsequently grouped into six categories in the hexagon (i.e., blue, UV‐blue, UV, UV‐green, green, and blue‐green) based on the relative excitations of the three types of bee photoreceptor (UV, blue and green). However, none of the plant species had bee‐blue petals, and thus all plant species were assigned to one of the other five color categories.

To assess whether floral traits are associated with visitation by vibrating bees, we calculated link temperature (Junker et al., 2010) for all of the plant species in each network. Link temperature quantifies whether the observed interaction frequency deviates from the expected interaction frequency based on a model of neutral interaction. If a link occurs more frequently than expected from random interaction, it is viewed as a “warm link,” whereas a link that occurs less often than expected is termed a “cold link.” Link temperature is calculated for each insect species separately, and we thus calculated the link temperature between each of the three vibrating bee species and all of the plant species in our communities. Link temperature (*T_ij_*) is calculated as:

$$T_{\mathrm{ij}}=\left[a_{\mathrm{ij}}‐\left(A_{i}\cdot \frac{A_{j}}{m}\right)\right]/A_{i}$$

Where *a_ij_* is the observed number of interaction between bee species *i* and plant species *j*. *A_i_* represents the total number of visits made by bee species *i* to all plant species in the community, and *A_j_* represents the total number of visits received by plant species *j* from all bee species (i.e., vibrating and non‐vibrating bees). The grand total of all interactions recorded between all plant species and all bee species in a community is given by *m*. Link temperature ranges from −1 to 1, where −1 indicates that a pollinator is avoiding a plant species and 1 indicates a pollinator is favoring a plant species (see Junker et al., 2010).

To test whether vibrating bees favor or avoid plant species based on visual traits, we implemented a linear mixed effect model using link temperature as response variable using the “lme4” package in R (Bates et al., 2015). We conducted stepwise backward model selection to determine which predictors and random effects to include in the final model (“step” function in “lmerTest” (Kuznetsova et al., 2017)). The model selection procedure included plant height, flower diameter, flower color group in bee space, and vibrating bee species as predictor variables. The model selection procedure also included plant species (because some plant species were present in multiple communities), site, and vibrating bee species identity as random effects. The final model included flower color predictor variable and plant species as random factor (Table 2), and the model was evaluated using Satterthwaite's method in “lmerTest” (Kuznetsova et al., 2017).

## 3 RESULTS

The six communities studied consisted of 26 plant species, of which 20 species offered pollen resources to bees (determined from observing pollinators on flowers). Communities showed both spatial and temporal turnover in plant species, based on flower densities recorded in 25–30 random 4 m^2^ plots at each site. In the first sampling session, the three communities showed 50.57% (SD = 13.76) similarity in plant species (Horn similarity ‐ Jost, 2007), and in the second session, communities showed 62.00% (SD = 20.01) similarity in plant species composition. Between sampling sessions (i.e., temporal similarity), communities showed 54.48% (SD = 16.71) similarity in plant species composition. The six communities thus showed sufficient turnover in plant composition for our purposes.

We recorded visits from 180 insect morphospecies, of which 66 morphospecies were bees. In total, we observed 7075 interactions between bees and pollen‐offering flowers across all six communities. Only three bee species visited *C*. *hyacinthoides*, that is, two *Anthophora* species and *Amegilla* cf. *niveata*, all of which used vibrations to extract pollen from *C*. *hyacinthoides*. Only *A*. cf. *niveata* visited *C*. *hyacinthoides* in all six communities. Across these six communities, we observed *Amegilla* cf. *niveata* collecting pollen from *Convolvulus capensis* (Convulvulaceae), *Cyanella hyacinthoides* (Tecophilaeaceae), *Roepera morgsana* (Zygophyllaceae), *Ornithogalum thyrsoides* (Hyacinthaceae), *Arctotis revoluta* (Asteraceae), *Euryops tenuissimus* (Asteraceae), and *Athanasia trifurcata* (Asteraceae).

The number of vibrating bee species visiting *C*. *hyacinthoides*, measured as interaction partner richness and diversity, was not influenced by the availability of other pollen sources (Table 1).

**TABLE 1:** Association between various measures of ecological specialization of *Cyanella hyacinthoides* and community composition (plant and insect)

Response	Predictor	Estimate	*z* or *t*	*p*
Interaction partner richness	Plant composition	0.6410	0.526	.60
	Vibrating bee abundance (log‐transformed)	0.3840	0.830	.41
Interaction partner diversity	Plant composition	1.1125	0.931	.42
	Vibrating bee abundance (log‐transformed)	0.4682	1.042	.37

Note

Plant community composition is represented by the second principal component of a PCA conducted on the abundances of all pollen‐offering plant species. Vibrating bee abundances are the abundances of *C*. *hyacinthoides's* pollinators. The association between the response and predictor variables were tested using a Poisson regression (for interaction partner richness) and linear regressions (for interaction partner diversity). No significant associations were detected.

**FIGURE 4:**
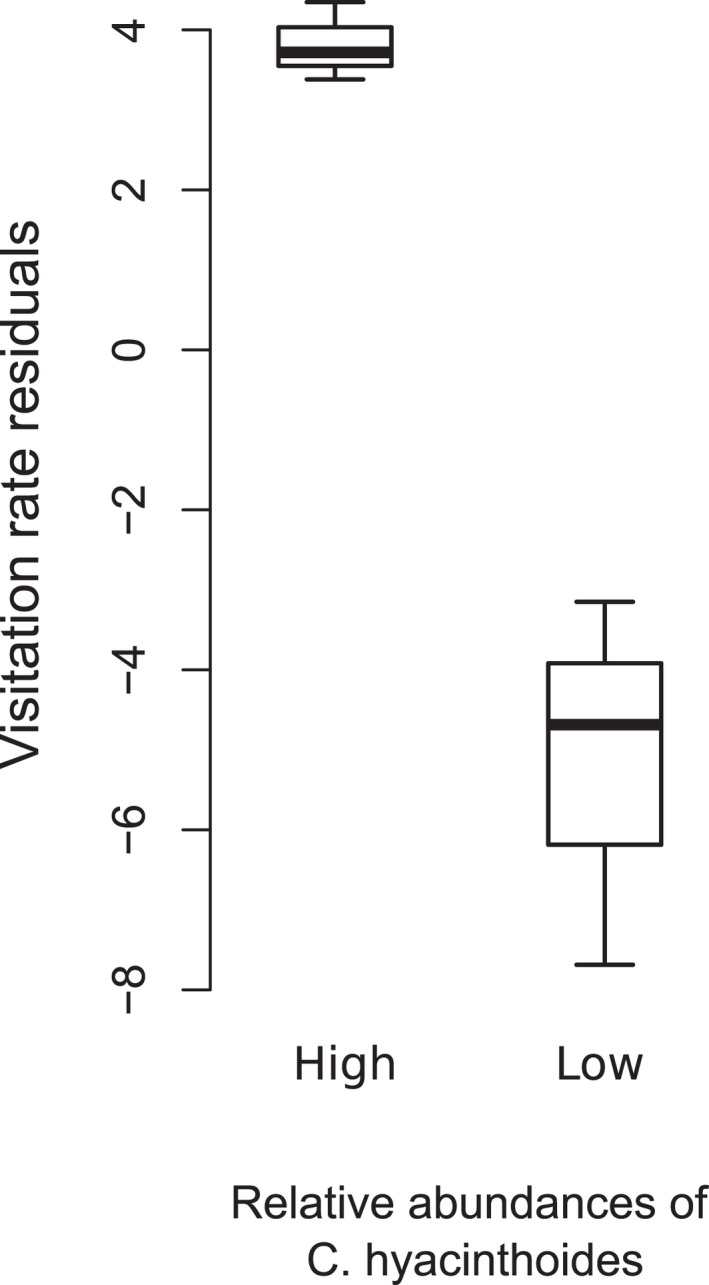
Paired *t*‐test comparing visitation rates to *C*. *hyacinthoides* (after adjusting for vibrating bee abundances) between communities with high and low relative *C*. *hyacinthoides* abundances (paired by site). Classification of communities within a site as high or low relative abundance were based on the second principal component of a PCA conducted on the abundances of all pollen‐offering plant species across all communities (see Figure 3)

The paired *t*‐test showed that *C*. *hyacinthoides* received more visits per flower in communities with high relative *C*. *hyacinthoides* abundances than communities with low relative *C*. *hyacinthoides* abundances, after controlling for vibrating bee abundances and site effects (*t* = 7.4006, df = 2, *p* = .02, Figure 4).

**FIGURE 5:**
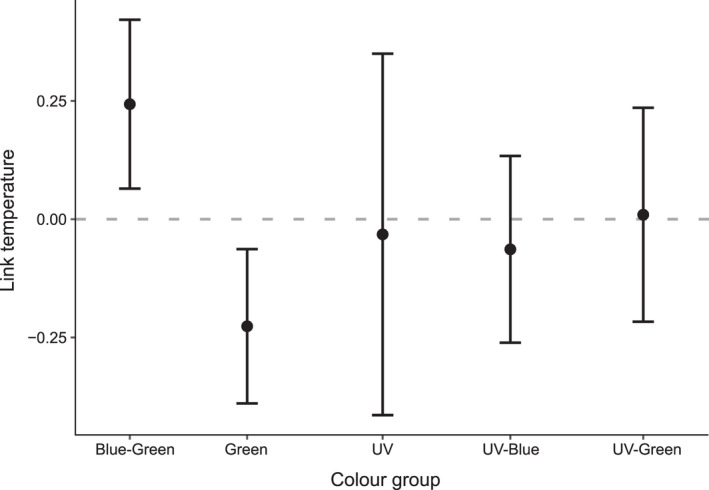
Differences in the link temperature between flowers of difference color categories (as perceived by bees). Plot shows predicted link temperature values for each color category based on a mixed effects model (link_temperature ~ colour_category + (1|plant_species)). High values of link temperature indicate that bees are preferentially visiting flowers of a particular color group, and low values indicate bees are avoiding a particular color group. Link temperature was calculated for all pollen‐offering plant species and the pollinators of *Cyanella hyacinthoides*

Our stepwise model selection procedure showed that color group was the only important predictor of link temperature (Table 2), and plant height, pollinator species, and flower diameter were thus excluded from the final model. Linear mixed effect modeling showed that link temperature was lower for flowers that fall into the green and UV‐blue hexagon sections compared to flowers classed as blue‐green (Figure 5, Table 2), suggesting that vibrating bees avoided green and UV‐blue flowers compared to blue‐green flowers.

**TABLE 2:** Backward model selection and subsequent linear mixed effect model results for features predicting link temperature of bees that use vibrations for pollen extraction

(a) Model selection using Satterthwaite's method (Type III ANOVA)			
Predictor variables	Sum of square	*F*	*p*
Plant height	0.0003	0.005	.94
Flower diameter	0.1168	2.325	.16
**Flower color group**	**0.7352**	**3.688**	.**03**
**Random effects**		**AIC**	***p***
Vibrating bee species		51.997	1.0
Site		49.997	1.0
**Plant species**		**55.584**	.**006**

(b) Linear mixed effect model			
Color group	Estimate	*t*	*p*
**Green**	**−0.4695**	**−3.805**	.**003**
UV	−0.2753	−1.279	.23
**UV‐blue**	**−0.3069**	**−2.260**	.**04**
UV‐green	−0.2337	−1.590	.14

Note

(a) Backward model selection was performed using the “step” function in the lmerTest package (Kuznetsova et al., 2017) with the link temperature of bees as response variable, the three predictor variables listed in the table below as predictor variables, and three random factors. Model selection was based on Satterthwaite's Type III ANOVA, and showed that flower color group (i.e., flower color category in the bee hexagon visual model) should be included as predictor variable and plant species as random factor in the final model. (b) We subsequently performed a linear mixed effect model with the link temperature of bees as response variable, flower color category as predictor variable, and plant species as random factor. Pairwise differences between groups were determined through *t*‐tests using Satterthwaite's method. The color group blue‐green is absorbed in the model. *p*‐values lower than .05 are indicated in bold.

## 4 DISCUSSION

We investigated the variation in pollination interactions (i.e., number of pollinator species and their visitation rates) of the buzz‐pollinated *Cyanella hyacinthoides* when it occurred in different co‐flowering communities. Although the number of bee species that visited *C*. *hyacinthoides* was not associated with the availability of more easily accessible pollen resources, the visitation rates of these bees to *C*. *hyacinthoides* were associated with the co‐flowering community composition. Specifically, *C*. *hyacinthoides* received more visits per flower when few other pollen resources were present in a community, and fewer visits per flower when many other pollen resources were available. Further, we show that bees exhibited non‐random visitation to flowers within these communities and avoided or preferred flowers with certain petal colors. Our results support the hypothesis that buzz‐pollinated flowers might be at a competitive disadvantage when more easily accessible pollen resources are abundant, particularly when the competitor species have similar floral traits.

### 4.1 *Cyanella* *hyacinthoides* is visited by few bee species

*Cyanella hyacinthoides* flowers were visited and vibrated by a small subset of the available bee species (4.5% of morphospecies). Only three of the 66 bee morphospecies were observed to use vibrations to extract pollen from *C*. *hyacinthoides*, with most of these visits made by *Amegilla* cf. *niveata*. Our results of pollination specialization in *C*. *hyacinthoides* aligns with findings from other buzz‐pollinated systems, where only a subset of bees in a community visited buzz‐pollinated taxa (Goldblatt et al., 2000; Mesquita‐Neto et al., 2018; Soares, 2021).

The three bee species that visited and vibrated *C*. *hyacinthoides* visited other plant species in the community to collect pollen. Although these three bee species collected pollen from a variety of plant families, they visited only a subset of the available pollen sources, suggesting that these polylectic bees exhibit floral preferences that do not only relate to pollen availability. Our results show that in our system, petal color likely mediates pollination interactions, and further study is required to determine which floral traits mediate pollination interactions in other buzz‐pollinated systems.

### 4.2 High relative availability of other pollen sources is associated with less buzz pollination

Our results demonstrate that per‐flower visitation rates of vibrating bees to *C*. *hyacinthoides* is dependent on the relative availability of more easily accessible pollen sources. In communities where flowers with easily accessible pollen were abundant, *C*. *hyacinthoides* received fewer visits per flower than in communities where *C*. *hyacinthoides* occurred in high relative abundances. Our results are supported by previous studies on flowers that are not poricidal but require complex handling behaviors. For instance, Stout et al. (1998) found that some species of complex flowers were at a competitive disadvantage in the presence of simple flowers with easily accessible resources. However, this was dependent on the identity of both the complex and simple flowered species, which suggests that additional floral traits are important in the foraging decisions of bees. In contrast to our results, Lázaro et al. (2013) showed that visitation to complex flowers did not change with heterospecific flower density but was rather related to pollinator abundances. Further, Lázaro et al. (2013) showed that seed set increased with an increase in both con‐ and heterospecific flower densities, suggesting facilitative interactions were prevalent among plant species. One potential reason for the contrasting results might be due to the identity of co‐flowering plant species and the rewards they offer (Stout et al., 1998). Although buzz‐pollinated species likely compete with other pollen‐offering flowers, the presence of nectar‐offering species in a community will potentially have facilitative effects on visitation to buzz‐pollinated flowers. The availability of nectar sources in a community might be particularly important for bees that use vibratile pollen extraction which requires high energy consumption (Pritchard & Vallejo‐Marín, 2020), and we might potentially expect the co‐occurrence of nectar sources to be a prerequisite for the occurrence of buzz‐pollinated species within a community, although this has not yet been investigated.

The reduced visitation rates to buzz‐pollinated flowers when unrestricted pollen resources occurred in high relative abundances can likely be attributed to the metabolic and/or learning costs associated with vibratile pollen extraction. The metabolic costs associated with vibratile pollen extraction is potentially more than 100 times as costly as resting metabolic rates (Dudley, 2002; Pritchard & Vallejo‐Marín, 2020; Vallejo‐Marín, 2021), and this might deter bees from visitation to buzz‐pollinated flowers when unrestricted pollen resources are readily available in a community. However, buzz‐pollinated flowers have been suggested to produce higher quality pollen than non‐buzz‐pollinated plants (Roulston et al., 2000), and this might incentivize bees to learn to effectively manipulate them when they occur in sufficient abundances. Further, the time required to learn to extract pollen from complex flowers might deter pollinators from visiting buzz pollinated flowers. Bees generally take longer to learn to extract resources from flowers that require complex handling behaviors than those that do not (Gegear & Laverty, 1998), but bees can remember flower‐handling techniques for long time periods (Chittka & Thomson, 1997; Keasar et al., 1996). Thus, if simple and complex flowers contain similar resources and are equally abundant, it is initially more costly for bees to visit flowers that require complex handling behaviors until these behaviors can be performed efficiently (Krishna & Keasar, 2018). The low visitation rates to *C*. *hyacinthoides* when their relative abundances are low might thus potentially be attributed to costs associated with learning to handle a rare and complex flower type. It would be interesting to assess whether handling times of *C*. *hyacinthoides* changes over the flowering season, and how handling times compare between *C*. *hyacinthoides* and other flowers in these communities.

Our results have significant implications for the reproductive ecology of *C*. *hyacinthoides* and other buzz‐pollinated plant species, and buzz‐pollinated plant species could potentially suffer reduced fitness in communities where they occur in low relative abundances. In non‐poricidal plant species, high abundances of co‐flowering conspecifics have been shown to increase pollinator visitation (Moeller, 2004; Rathcke, 1983), as well as pollen removal and deposition (Duffy & Stout, 2011), due to the increased size of the floral display. Although an increase in plant density can increase per‐flower visitation rates (e.g., Duffy & Stout, 2011), this will likely saturate at high plant densities due to pollinator limitation, and visitation rates are likely to decrease at higher plant densities. Our results contrast with those of Johnson et al. (2012) and Stout et al. (1998) who showed that complex flowers received more visits per flower when they occurred in low densities than high densities. However, our sampling design did not include communities with extremely high flower densities (densities ranged from 1.08 to 11.23 *C*. *hyacinthoides* flowers per m^2^ and from 0.15 to 2.06 *C*. *hyacinthoides* plants per m^2^), and thus we cannot rule out that the visitation rates might decrease at higher densities (e.g., Zimmerman, 1980).

Although we were able to utilize the natural spatial and temporal turnover in plant community composition of the CFR to evaluate the influence of relative co‐flowering plant abundances on buzz‐pollination interactions, we were not able to evaluate the effects of absolute abundances. Controlled field experiments (e.g., potted plants in arrays) would be required to determine whether our results hold when both the relative and absolute abundances of the co‐flowering community is low.

### 4.3 Bees preferred and avoided flowers with certain colors

Our understanding of the motivation of bees to visit poricidal flowers within a community of flowering plants remains limited. Previous work by Mesquita‐Neto et al. (2018) found that different buzz‐pollinated plant species within a community in Brazil were visited by different subsets of vibrating bees, which suggests that floral traits other than poricidal anthers might be important in enabling or limiting visitation to buzz‐pollinated flowers. Here, we show that pollination interactions in our study are partly mediated by the visual signals of *C*. *hyacinthoides* in this system. The pollinators of *C*. *hyacinthoides* visited plant species with bee‐blue‐green reflective petals more frequently than those that primarily reflect bee‐UV‐blue or bee‐green. In contrast to our results, previous work has shown that some bee species have innate preferences for bee‐UV‐blue (Giurfa et al., 1995) and for bee‐green (Giurfa et al., 1995). Flower color, however, is a complex multi‐faceted signal (Bukovac et al., 2017; Ng et al., 2018), and other visual aspects such as achromatic contrast or color patterning might also mediate pollinator foraging decisions.

Further, although *C*. *hyacinthoides* flowers reflected bee‐blue‐green, eight of the 26 species reflected this color, and thus *C*. *hyacinthoides* did not provide a unique color signal. We observed, however, that *C*. *hyacinthoides* flowers emitted a strong scent, and scent might be an important mediator in these buzz‐pollination interactions, similar to what has been shown for other buzz‐pollinated taxa (Solís‐Montero et al., 2018; Vega‐Polanco et al., 2020).

### 4.4 Conclusions and future directions

Our work represents one of the first studies on the community ecology of buzz‐pollination interactions, and we show that the co‐flowering community of pollen‐offering species influenced visitation to a buzz‐pollinated species. Bees preferentially used vibratile pollen extraction when flowers with poricidal anthers occurred in high relative abundances, suggesting that it might be costly for bees to seek them out among other species when such flowers are rare. A promising avenue for future work encompasses determining whether this cost relates to time spent flying between low‐density flowers, the energy cost of using vibrations for pollen extraction, or whether cognitive constraints limit the number of flower handling behaviors bees perform on a foraging bout. In line with this, quantifying the amount of pollen collected per unit energy spent on buzz‐pollination compared to collecting pollen from easily accessible but unreliable pollen sources, remain to be investigated and will provide valuable insight into the evolution of buzz‐pollination. We also show that flower color is important in mediating pollination interactions in these communities, and future work should investigate whether pollen‐offering plant species with the same flower color have facilitative or competitive effects on buzz‐pollination interactions. Although we only explored the influence of the co‐flowering pollen‐offering species, the availability of flowers that offer nectar resources will likely also influence buzz‐pollination interactions. Bees collect both pollen and nectar resources, and if bees prefer collecting nectar from particular plant species, the presence of these species in a community or in a patch might facilitate visitation to flowers with poricidal anthers. Additionally, these nectar sources might be important in providing bees with the necessary sugar (i.e., energy) to sustain vibratile pollen extraction.

## CONFLICT OF INTEREST

The authors have no conflict of interest to declare.

## AUTHOR CONTRIBUTIONS

**Jurene E. Kemp:** Conceptualization (lead); Data curation (lead); Formal analysis (lead); Funding acquisition (equal); Investigation (equal); Methodology (lead); Project administration (lead); Visualization (lead); Writing – original draft (lead); Writing – review & editing (equal). **Francismeire J. Telles:** Conceptualization (supporting); Data curation (supporting); Investigation (equal); Methodology (supporting); Writing – review & editing (supporting). **Mario Vallejo‐Marín:** Conceptualization (supporting); Formal analysis (supporting); Funding acquisition (equal); Investigation (supporting); Project administration (supporting); Visualization (supporting); Writing – original draft (supporting); Writing – review & editing (equal).

## Data Availability

Plant density and plant‐pollinator interaction data are available on Dryad (https://doi.org/10.5061/dryad.3tx95x6j4).
